# Voice restoration following total laryngectomy by tracheoesophageal prosthesis: Effect on patients' quality of life and voice handicap in Jordan

**DOI:** 10.1186/1477-7525-6-26

**Published:** 2008-03-28

**Authors:** Abdelrahim Y Attieh, Jeff Searl, Nada H Shahaltough, Mahmoud M Wreikat, Donna S Lundy

**Affiliations:** 1 Speech Rehabilitation Department, Royal Rehabilitation Center, King Hussein Medical Center, Amman, Jordan; 2 Department of Hearing and Speech, University of Kansas Medical Center, Kansas, USA; 3 Department of Otolaryngology, King Hussein medical Center, Amman, Jordan; 4 Department of Plastic & Reconstructive Surgery, Royal Rehabilitation Center, Director, Amman, Jordan; 5 Department of Otolaryngology, University of Miami School of Medicine, Florida, USA

## Abstract

**Background:**

Little has been reported about the impact of tracheoesophageal (TE) speech on individuals in the Middle East where the procedure has been gaining in popularity. After total laryngectomy, individuals in Europe and North America have rated their quality of life as being lower than non-laryngectomized individuals. The purpose of this study was to evaluate changes in quality of life and degree of voice handicap reported by laryngectomized speakers from Jordan before and after establishment of TE speech.

**Methods:**

Twelve male Jordanian laryngectomees completed the University of Michigan Head & Neck Quality of Life instrument and the Voice Handicap Index pre- and post-TE puncture.

**Results:**

All subjects showed significant improvements in their quality of life following successful prosthetic voice restoration. In addition, voice handicap scores were significantly reduced from pre- to post-TE puncture.

**Conclusion:**

Tracheoesophageal speech significantly improved the quality of life and limited the voice handicap imposed by total laryngectomy. This method of voice restoration has been used for a number of years in other countries and now appears to be a viable alternative within Jordan.

## Background

Total laryngectomy results in physical and functional changes that can affect the emotional well-being and some of the most basic functions of life, including breathing, swallowing, and communication [1]. Proper education and counseling from health care providers can help patients to adapt to the changes related to the procedure, but, even with strong counseling, the changes to communication and other body functions are often overwhelming for individuals and their families [2]. After total laryngectomy, the person breathes through a stoma in the neck which may elicit a negative reaction from the patient and from others [3]. Additionally, re-routing of breathing through a stoma often results in increased mucus production, coughing, and possibly extraneous noise during breathing. Other common issues reported after total laryngectomy include dysphagia [4], change in taste and smell [5], and neck and shoulder movement problems [6]. Repeated visits to the hospital, job loss, and worries of cancer recurrence can add to the psychological burden on patients and families [7]. Difficulties in one or several of these areas could negatively impact a person's perceived quality of life.

Although laryngectomy can result in a number a changes, the alterations to voice and speech production are perhaps the most obvious and the rehabilitation process focuses heavily on re-establishing functional communication. In general, patients who undergo total laryngectomy experience a decreased quality of life compared to patients after partial laryngectomy or healthy individuals [8-10]. While the alteration to speech is not the only contributor to reduced quality of life, it is generally considered a major factor [10].

Successful restoration of voice and speech after total laryngectomy is dependent on a number of variables. Access to knowledgeable and competent physicians and speech therapists is one basic necessity. In some regions of the world, health care providers are clustered in major population centers. This can leave those in outlying areas at a disadvantage in the rehabilitation process if they are unable or unwilling to travel for their care. This is the case in Jordan where most speech therapists are located in the capital. Those individuals living in remote areas of the country tend to be in a lower socioeconomic class and the financial burden for traveling to receive health care is often difficult to overcome. Although the literacy rate for Jordan as a whole is quite high in those under age 60, illiteracy in the elderly can be an issue. As noted below, one third of the Jordanian laryngectomees in this study were unable to read. While this does not preclude successful alaryngeal speech rehabilitation, it can make the process more challenging in that written materials and instructions cannot be used as effectively. As Eadie and Doyle [11] indicated, both education and socioeconomic status could influence a person's degree of involvement in their own care and their ability to access services. Other societal characteristics could conceivably impact the rehabilitation process as well, although these have not been heavily investigated. For example, cultural views of disfigurement and disability may serve to isolate an individual. As in many parts of the world, in regions of lower SES in the Middle East, there is a certain degree of social stigmata and discrimination of individuals who are disabled in some way, and perhaps more so if the disability is readily visible or apparent as occurs following total laryngectomy. Significant alterations to a person's ability to work or support a family because of a disease or condition might substantially alter an individual's role within a family or culture. This may be more applicable in rural areas of Jordan where men are more likely to be the primary head of the household.

Schuster et al [10] and others [12-14] have indicated that an individual's social adjustment, general coping skills and overall well-being may impact the success of alaryngeal speech rehabilitation. The extent to which an individual copes and adjusts to living without a larynx is presumably influenced by many variables, some of which are inherent to the individual such as their general attitude toward stress, while others might be more broadly referred to as cultural (as described above). In addition, there has been some speculation in the literature that quality of life might be differentially impacted by the method of alaryngeal communication that a person uses, although more work is needed in this area [15]. Each method of speech has disadvantages. Esophageal and electrolaryngeal speech have been part of the rehabilitation process for many years around the world. Specific data about usage patterns within Jordan and other parts of the Middle East are not available in the literature. However, clinical observations within our clinic suggest that neither esophageal nor electrolaryngeal speech are commonly adopted within the Jordanian laryngectomee community. Buccal speech has been more commonly observed although the reasons for this are not readily apparent. Lack of available speech therapists to train the more traditional communication methods in some parts of the country and/or reduced patient access to services within the capital may be the primary limits. Esophageal and electrolaryngeal speech do also have some inherent limitations that may have been unacceptable to most Jordanian laryngectomees, just as they have been for some larygnectomees in other parts of the world. For example, esophageal speech is generally characterized by low pitch [16], reduced loudness [17], altered voice quality (glottal fry, hoarse, rough, breathy have all be identified) [18], limited number of syllables per breath [19], and a lower rate of acceptability by listeners [19]. In addition, our experience has been that in Jordan, esophageal speech may be viewed as rude because it is similar to a burp or spitting in a listener's face. Electrolaryngeal speech has been described as mechanical sounding and does require the use of one hand during communication to hold the device; additionally, it often is the least preferred method of alaryngeal communication by listeners and clinicians [18,20].

Tracheoesophageal speech is the newest alaryngeal communication option [21] and it has provided patients with a communication means that more closely approximates normal laryngeal voice in terms of air supply, duration, loudness, and inflectional patterns [22,23]. For some TE speakers, a voice that more closely approximates laryngeal speech may be reflected in ratings of quality of life and degree of voice handicap that are more similar to non-laryngectomized speakers, although this remains to be demonstrated more definitively [11,24]. The availability of the TE puncture procedure has been increasing in Jordan over the past several years, but outcome data are lacking. One approach for documenting treatment outcomes is to assess the patient's perception of their quality of life before and after using a specific rehabilitative technique. This is usually assessed through disease-specific "quality of life" measures that are confirmed with *a priori *expectation [25]. A disease-specific measure asks questions about the impact of a particular disease or condition on various aspects of a person's quality of life. In contrast, a general health-related quality of life tool takes into account a broad range of health issues and their impact on a person's life. Quality of life instruments are often used to evaluate treatment effects from the patients' point of view [10]. Such tools adopt the needs-based model of quality of life, which postulates that life gains quality from the ability of individuals to satisfy their own needs [26]. In the area of head and neck cancer, one commonly used instrument is the University of Michigan Head & Neck Quality of Life Instrument (HNQOL) developed and validated by Terrell et al [27] and used with a number of cultures and languages [10,28,29]. Several studies have utilized this instrument to assess the quality of life of laryngectomized individuals after prosthetic voice restoration [10,11,27-30], although these studies have been largely restricted to North American and European populations. To the authors knowledge there have not been any reports from Arabic-speaking Middle Eastern countries.

The HNQOL contains 20 five-choice Likert questions that are used for scoring under four domains to assess the quality of life: communication (4 items), eating (6 items), pain (4 items), and emotions (6 items). It also assesses global satisfaction with treatment. Another internationally used measure is the SF-36 questionnaire which was translated and validated into Arabic by Abdulmohsin et al [31] and Coons et al [32].

The degree of limitation or handicap resulting from the voice of laryngectomized patients using TE speech can be assessed with the Voice Handicap Index (VHI) [33]. This instrument was developed by Jacobson et al [34] and is used for measuring the psychosocial handicapping impact of voice disorders. It can also be used for measuring the therapeutic outcome of voice therapy, as well as rating the severity of the voice problem [33,35]. The VHI covers three domains, namely functional, physical, and emotional. Each domain is addressed by 10 questions with a 5-choice Likert response (0 – 4). The application of such an instrument with laryngectomized patients can help document the influence that a particular therapeutic intervention, such as implementation of TE speech, has on the degree of vocal handicap experienced by an individual. According to Schuster et al [36], both health-related quality of life and voice handicap are not affected in a group specific way as shown by a wide range of collected data. They concluded that a quality of life instrument should be combined with the VHI in order to describe the individual aspects of the laryngectomee's well-being. It should be emphasized that the University of Michigan HNQOL contains only four items in its communication domain, while all three subtests of the VHI 30 items survey only communication dimensions.

The purpose of this study was to compare the quality of life and degree of voice handicap of laryngectomized Jordanian patients before and after successful TE voice restoration. Such a report on Jordanian speakers has not yet appeared in the literature but is of increasing importance as the number of TEP procedures increases in this country. The null hypothesis was that there would be no difference in scores before and after TE voice restoration. The relationship between the ratings of quality of life and ratings of the degree of voice handicap also was of interest. The null hypothesis regarding this relationship was that changes in the voice handicap would not be associated with changes in the quality of life of laryngectomized patients.

## Methods

Twelve male Jordanian laryngectomized patients using Blom-Singer (Inhealth^®^) voice prostheses as their primary mode of communication were studied. Each patient, or a family member, was asked to complete a general information form to gather biographical and medical history. Table 1 includes demographic and other descriptive data for the group of participants. The Committee of Medical Research Ethics approved the study and all subjects, or a family member, provided informed consent.

**Table 1:** Subjects of the study. (all males). TL refers to total laryngectomy and TE refers to tracheoesophageal.

Patients	Age	Interval Between TL and TE Puncture	Time post TE for Second Survey Administration	Previous means of communication	Radio Therapy sessions	Education level
AA	61	1;1 yrs	9 mo.	TEP done abroad	33	lawyer
EA	62	1;8 yrs	9 mo.	TEP done abroad	none	Illiterate
AB	58	1 mo.	9 mo.	Non-vocal	none	Illiterate
FF	69	16;1 yrs	6 mo.	Buccal speech	30	High school
SR	51	8 mo.	5 mo.	Buccal speech	35	High school
FR	62	1;9 yrs	8 mo.	TEP done in the private	32	BA
HM	66	5 mo.	3 mo.	Esophageal speech	25	Illiterate
JA	35	1;9 yrs.	9 mo.	Buccal speech	none	High school
RA	74	8 mo	6 mo.	Electrolarynx	30	High school
MM	67	1;9 yr	9 mo.	Esophageal speech	30	M. Sc. engineering
MH	64	1;8 yrs	7 mo.	Buccal speech	35	Junior high school
MJ	69	1;2 yrs	9 mo.	Buccal speech	none	Illiterate
Summary	Mean: 61.5 S.D.: 10.2	Mean: 2;4 S.D.: 4;4	Mean: 7.4 S.D.: 2.0	Buccal = 42% Prior TEP = 25% Esophageal = 17% Electrolaryngeal = 8% Non-vocal = 8%	Radiation: 67%	illiterate = 33% high school = 33% graduate school = 17%, undergraduate = 8%, junior high = 8%,

Prior to the study, none of the patients were using TE speech for communication, although three had previously tried it but had allowed the puncture to close. All of them had the TE puncture done as a secondary surgical procedure. In the interim between the time of the total laryngectomy and the time of the TE puncture, subjects used either buccal, electrolarynx, or esophageal speech for communication. Prior to the TE puncture, each patient, with the help of a clinician or a family member, completed an Arabic translation of the University of Michigan Head and Neck Quality of Life instrument (HNQOL) and the Voice Handicap Index (VHI). Each subject completed these two surveys a second time three to nine months following the TE puncture procedure. At this second data collection time, all subjects were using TE speech functionally and were judged to have 'average' to 'good' TE speech in terms of intelligibility and loudness as judged by their families.

For the data analysis, pre- to post-TEP changes were assessed for the subcomponent scores for the HNQOL and the VHI, respectively, and also for the total scores on each instrument. Paired t-tests were used for pre-post comparisons. There were 5 paired comparisons from the HNQOL. A 0.05 alpha level was shared across this family of five comparisons so that a probability level of 0.01 or smaller was necessary to consider a difference to be statistically significant (i.e., 0.05/5 = 0.01). Likewise, the alpha level was adjusted across the family of four paired comparisons for the VHI data so that a probability level of 0.0125 was needed to reach statistical significance. In addition, the total scores on both instruments were correlated using Pearson Product Moment Correlation.

## Results

**Figure 1:**
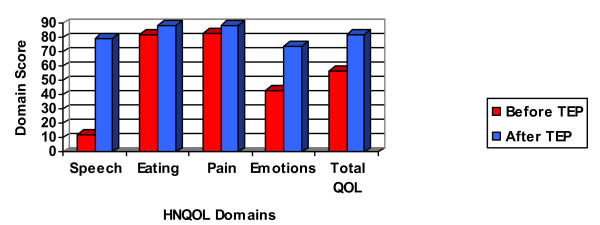
Descriptive gains of our cohort on total and various QOL domains before and after TE speech.

Complete data sets were available from all patients. Tables 2 &3 and Figure 1 show the patients' scores before and after prosthetic voice restoration on each domain of the HNQOL and the VHI, respectively. Paired t-tests for the total and subtests of the HNQOL (Table 4) show that the patients' quality of life was significantly improved in the communication (p ≤ 0.001), emotions (p = 0.001), and the total QOL score (p ≤ 0.001). As a group, the 'communication' domain score was 84% higher post-TEP (79.2) than it was pre-TEP (12.3). The 'emotion' domain was 31% higher post-TEP (74) compared to pre-TEP (43.4). Finally, the 'total' score on the HNQOL was 25% higher post-TEP (82.3) compared to the pre-TEP rating. The 'pain' and the 'eating' domains did not differ significantly from pre- to post-TEP (p > 0.01, respectively).

**Table 2:** Group scores before and after prosthetic voice restoration on each domain of the H&N QOL. Com, Eat, Pain &, Emo, refer to Communication, Eating, Pain, & Emotions subtests, respectively. Tota refer to total score. Numbers 1 & 2 refer to before & after voice restoration, respectively.

Patients	Com1	Com2	Eat1	Eat2	Pain1	Pain2	Emo1	Emo2	Tota1	Tota2
Mean	12.3	79.2	82.3	88.5	82.8	88.5	43.4	74.0	56.9	82.3
Standard Deviation	15.2	18.5	14.2	11.8	15.3	13.3	22.7	28.2	12.5	14.5
Range	0–56	37–100	54–100	67–100	56–100	63–100	13–100	29–100	43–79	54–99

**Table 3:** The patients' scores before and after prosthetic voice restoration on each domain of the VHI. *Funct*, *Phys*, &*Emot *refer to Functional, Physical, & Emotional domains. VHI refers to the total score. Numbers 1 & 2 refers to before & after voice restoration, respectively.

Patients	Funct1	Funct2	Phys1	Phys2	Emot1	Emot2	VHI 1	VHI 2
Mean	36	14	30	13	27	9	93	36
Standard Deviation	4	9	8	6	11	9	18	21
Range	28–40	0–28	15–40	5–26	6–40	1–30	59–120	8–77

**Table 4:** Paired t-test statistics for H&N QOL instrument

	Paired Difference							
				
QOL pairs	Mean Difference	SD	Standard Error of Mean	95% Confidence		t	df	P
							
				Lower	Upper			
Comm1 – Comm2	-66.83	20.44	5.90	-79.83	-53.85	-11.3	11	<0.001
Swal1 – Swal2	-6.25	10.28	2.97	-12.78	0.28	-2.1	11	0.059
Pain1 – Pain2	-5.73	9.41	2.72	-11.71	0.25	-2.1	11	0.059
Emot1 – Emot2	-30.56	23.53	6.79	-45.50	-15.61	-4.5	11	0.001
QOL1 – QOL2	-25.42	10.50	3.03	-32.09	-18.74	-8.4	11	<0.001

**Figure 2:**
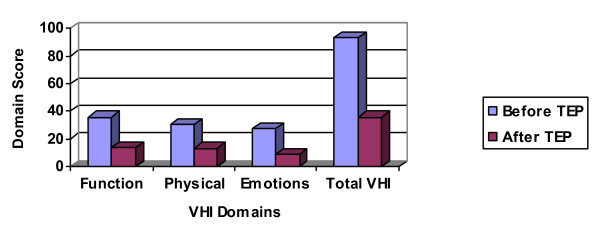
Descriptive gains of our cohort on total and various VHI domains before and after TE speech.

As indicated in Table 5 and Figure 2, all four paired comparisons for the VHI data (the 3 subtests and the total score) were statistically significant using the adjusted alpha level of 0.0125. For each subsection of the VHI, participants reported less voice handicap following TE voice restoration. The post-TEP ratings of handicap were 39% lower for the 'function' subscale, 43% for the 'physical,' 33% for the 'emotional,' and 39% for the 'total' compared to the pre-TEP ratings.

**Table 5:** Paired t-test statistics for the VHI instrument. F, P, & E refer to Function, Physical, & Emotions subtests of the VHI, respectively.

	Paired Difference							
				
VHI pairs	Mean Difference	SD	Standard Error of Mean	95% Confidence		t	df	P
							
				Lower	Upper			
F1 – F2	21.92	10.09	2.91	15.51	28.32	7.53	11	<0.001
P1 – P2	17.00	8.83	2.54	11.39	22.61	6.67	11	<0.001
E1 – E2	18.42	10.46	3.02	11.77	25.06	6.10	11	<0.001
VHI1 – VHI2	57.33	25.58	7.38	41.08	73.59	7.76	11	<0.001

Although not part of the planned analysis, inspection of the participant pool indicated that the interval between surgery and the post-TEP data collection varied widely from 1 month to 16 years. In order to allow more informed interpretation of the pre- to post TEP QOL data, more information was sought regarding the impact that "time post laryngectomy" might play in the QOL ratings. Pre- to post-TEP difference scores were calculated for each subsection and total score on the HNQOL and VHI, respectively. The interval (in months) between total laryngectomy and administration of the post-TEP QOL surveys also was then correlated to the difference scores (Table 6). None of these correlations was statistically significant. The lack of significant relationships suggested that the magnitude of change on QOL subsections and total scores was not closely related to how long ago they had their laryngectomy.

**Table 6:** Pearson product moment correlation coefficients and associated probability values for the interval duration between total laryngectomy-to-post-TEP QOL ratings and the difference scores for the subsections and total scores on the HNQOL and VHI, respectively.

	*r*-value	*p*-value
**HNQOL**		
Communication	-.491	.105
Eating	-.133	.681
Pain	-.010	.976
Emotion	-.226	.480
Total	-.380	.224
**VHI**		
Function	.354	.259
Physical	.147	.650
Emotion	.318	.314
Total	.320	.311

A *Pearson Product Moment Correlation *coefficient was calculated to evaluate the relationship between the change in VHI total score and the change in the HNQOL total score. The *r *– value of 0.523 was not statistically significant (*p *= .081). Because of the interest in describing the impact of changes in speech (i.e., introduction of TE speech) on quality of life, one other correlation was calculated. There was a strong and statistically significant correlation between the VHI total score and the 'communication' domain of the HNQOL (*r *= .841, *p *= 0.001).

## Discussion

This study is the first report of QOL and voice handicap for Jordanian speakers following total laryngectomy. An important component of this study was that pre- and post-TE puncture quality of life scores were gathered for each participant to better assess the impact that establishment of TE speech might have on this group of individuals. TE puncture has been available for over 25 years in some parts of the world. However, the procedure is only now becoming more common in Jordan. The expansion of a therapeutic option into a particular region of the world should be accompanied by investigations regarding outcomes because the local professional resources (medical, speech pathology, etc.), cultural characteristics, physical environment, and so forth, might have influence on the viability of the speech option within that region.

Although the number of subjects is small, this study afforded the opportunity to make preliminary observations about the pattern of alaryngeal speech usage within Jordan. Buccal speech was the most common form of alaryngeal speech among the 12 participants prior to undergoing TE puncture. A larger sample is needed to confirm whether the current group is representative of the practice pattern within Jordan. However, our clinical experience in Jordan is consistent with the finding that buccal speech is used frequently, although the reasons for this are not clear. As noted earlier, esophageal speech may be considered offensive to some because it is viewed as "burping" which could be insulting to the listener. Likewise, the electrolarynx is often not viewed favorably, particularly in rural regions or in populations with lower SES because it marks the speaker as unusual (one speaker received the negative label as "the one with the buzzer"). Presumably, reduced access to speech training or reduced willingness to go through the training process for either esophageal or electrolaryngeal speech also could have influenced the speech option that ultimately was adopted prior to this study.

As in many parts of the world, access to speech pathologists capable of training alaryngeal speech may be limited within the country. Several of the participants came from remote areas where formal speech training was not available. One possibility is that buccal speech is preferred in this culture, although we have no direct evidence for this, nor do we suspect that is the case. In general, buccal speech has been discouraged by clinicians in Europe and North America because of its unusual quality, limitations in loudness and pitch manipulation, and restricted phrase lengths [37]. However, it may be that buccal speech is more easily acquired than esophageal speech for an individual who is left without formal alaryngeal speech rehabilitation. Additionally, a number of the participants had low socioeconomic status that may have imposed financial restrictions (either for payment of services, or travel to receive services) limiting the possibility of learning one of the more traditional alaryngeal speech options such as esophageal or electrolaryngeal speech.

**Figure 3:**
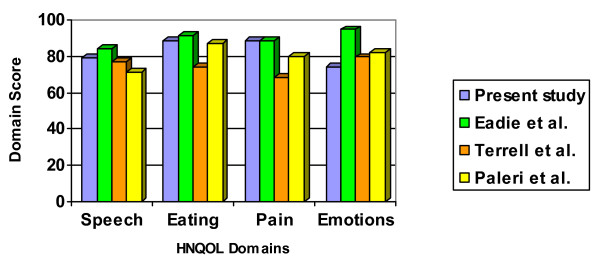
Descriptive comparisons of the QOL domains between the present study and some other published studies[11,28,38].

As shown in Figure 3, the HNQOL scores post TEP in the present study were comparable to those from Eadie & Doyle [11] with the exception of values for the 'emotion' domain which were approximately 20 points lower (i.e., 'worse') in the current study. With one exception (again, the 'emotion' domain), the HNQOL scores for the current participants were comparatively higher than the long-term QOL reported by patients in studies by Terrell et al [38] and Paleri et al [28]. Terrell et al and Paleri et al both included individuals using any of the three primary alarygneal speech options. Inclusion of individuals using eletrolaryngeal and esophageal speech may have lowered the group mean scores for the total score on the HNQOL. Electrolaryngeal speakers, for example, reportedly have rated their quality of life as being worse than TE speakers [39]. The scores on the emotion domain of the HNQOL in the current subjects were, however, lower than values for the 'emotion domain reported in Terrell et al, Paleri et al, and Eadie & Doyle. The reason(s) for the notably lower score on the 'emotion' component is not readily discernable from the current study, although some speculation is possible based on our clinical observations in Jordan.

Cultural attitudes toward illness and disability may play some role. Several of the participants were illiterate and from lower socioeconomic group. Based on informal comments, they felt fairly isolated in their home community. In more remote parts of the country where illiteracy rates are higher and contact with medical professionals is less likely, there is little understanding of what total laryngectomy is, why the person's speech is changed, or what the available options are for communicating after the procedure. Although pre-operative counseling is used to help educate patients and families, they often do not retain all of the information or they are unable to pass this information along sufficiently to those in their local community. In addition, fears of cancer recurrence seem particularly high which may partly be depressing the QOL ratings in the emotion domain. Additionally, the isolation and emotional difficulties could be related to difficulty with communication in at least some cases. For example, speakers MM and EA both had pre-TEP scores indicating significant reduction in quality of life in the 'communication' and the 'emotion' domains. Following TEP, not only did the 'communication' domain score increase markedly, but so did the 'emotion' domain score. Although cause can not be determined, it seems reasonable to speculate that improved communication may be at least partly responsible for the improvement in the emotion score. However, there are also examples where significant changes in 'communication' domain scores following TEP were not accompanied by improvements in emotion scores. Participant AA is perhaps the best example of this. He had the lowest (i.e., 'worse') score on the 'emotion' domain prior to the TEP and also the lowest 'communication' domain score (tied with two others). Following the TEP, the 'communication 'domain' score increased substantially (from 0 to 81.25) indicating a marked improvement in quality of life related to communication. However, AA's 'emotion' domain score following the TEP, although increased from pre-TEP, remained as the lowest score among the group and it was more than 50% less than the group mean score.

The significant improvement in quality of life related to communication following TEP was not completely unexpected. Based on the pre-TEP 'communication' domain scores on the HNQOL and the high degree of voice handicap reported in the pre-TEP VHI instrument, it seems reasonable to conclude that this group of 12 speakers were experiencing a very high degree of difficulty in their life associated with communication at the start of this study. Nearly half of the group was using buccal speech and these participants informally indicated that they had essentially no useable method of verbal communication for daily activities. One patient (FF) went for over 15 years with buccal speech that was quite poor, leaving him isolated, and by his report, lonely, prior to his TEP. With the exception of one esophageal speaker (HM), the remaining esophageal speaker, artificial larynx user, and the group who had previously tried a TEP also reported extremely limited communication abilities prior to the TEP. Given the relatively dramatic change from almost no useable speech to functional speech following TEP for a sizeable portion of the current group, it is not surprising that communication scores in particular and quality of life and handicap scores in general were significantly improved. Establishing TE speech as a functional communication option was not only evident in their informal comments to the investigators but is also reflected in the change in all four of the VHI scores and HNQOL 'communication' score. All of the subjects in the current study who had some prior form of alaryngeal speech indicated that their newly established TE speech more closely resembled their pre-laryngectomy speech than did their prior esophageal, buccal or electrolaryngeal speech.

One could argue that the additional time post-total laryngectomy that was encompassed within this study (on average, 7.4 months from the TE puncture to the second administration of the quality of life measures) might have contributed to further adjustments to living without a larynx and, subsequently might have contributed to improvements in quality of life ratings. That is, the individuals might have simply had more time to integrate back into society and adapt to the changes in their life regardless of whether TE speech was introduced. However, all but two of the speakers were more than 6 months post-larygnectomy, and two-thirds of the group was a year or more post-laryngectomy, prior to the start of their participation in this study. They had all stabilized medically prior to the start of the study and their ratings on the swallowing and pain subsections of the HNQOL were quite high in the pre-TEP data collection period supporting the notion that other functions besides communication were relatively less impacted at that point. Introduction of TE speech was the primary change in status for this group of individuals and there was a substantial change in perceived quality of life. In addition, Schuster et al [10] and Eadie & Doyle [21] did not find a significant correlation between scores on quality of life instruments and the period of time since laryngectomy.

The group data indicate a positive change in quality of life and voice handicap ratings post-TEP. Inspection of the data for individual speakers also supports this conclusion although there is a fair amount of variation in the degree of handicap, impact on quality of life, and the amount of change in these measures following TE voice restoration. For some individuals, improvements in QOL and degree of handicap may have been constrained by some of the more routine difficulties associated with TE speech. For example, most of our patients were unable to purchase the hands-free heat and moisture valve, and then were annoyed by the need to use their hands to close their stoma for speech. Many of them have to come back to clinic frequently for replacement of the prosthesis due to leakage problem. One individual, although a proficient TE speaker, did not show much change in his quality of life and he specifically commented that he felt the physical disfigurement following surgery was causing others to avoid him. Establishment of functional TE speech apparently was not enough to counteract the negative impact on his quality of life from the physical disfigurement.

## Conclusion

The present study indicated that the quality of life and degree of voice handicap of the laryngectomized individual in Jordan could be improved by providing a functional means of communication in the form of TE speech. In this group of 12 Jordanian males, the use of TE voice appeared to be associated with a decrease in the voice handicap.

The healthcare system in Jordan provides a wide range of services for cancer patients. However, voice rehabilitation following total laryngectomy is restricted to Amman, the capital, military medical facilities, the King Hussein Cancer Center, and few private clinics of otolaryngology. This centralization of services may impose restrictions on the availability and accessibility of alarygneal speech services to those living outside this area. This study demonstrates a positive, short-term outcome related to quality of life once TE speech was established. Long-term outcome data will be important to pursue given the service restrictions and cultural issues that could place burdens on successful alaryngeal speech rehabilitation.

## List of abbreviations

HNQOL stands for the Head & Neck Cancer-Related Quality of Life. SF36 stands for the Short Form 36-item Health Survey. TE stands for Tracheo-esophageal. TEP stands for Tracheo-esophageal Voice prosthesis. TL stands for Total laryngectomy. VHI stands for the Voice Handicap Index.

## Competing interests

The author(s) declare that they have no competing interests.

## Authors' contributions

All the authors designed the study and revised the manuscript for intellectual content. AAwas also responsible for analyzing and interpreting the data and drafting the manuscript. DLfirst introduced the technique of TE speech within Jordan. JSrevised the cultural influences of such technique. NSalso worked on problem solving of TEP complications which affected QOL. MWwas responsible for the conception of the study. All authors have read and approved the final manuscript.
